# Using environmental health officers’ opinions to inform the source attribution of enteric disease: further analysis of the “most likely source of infection”

**DOI:** 10.1186/1471-2458-14-1258

**Published:** 2014-12-11

**Authors:** Anna Lukacsovics, Andrea Nesbitt, Barbara Marshall, Rod Asplin, Jason Stone, Glen Embree, Matt Hurst, Frank Pollari

**Affiliations:** Department of Population Medicine, Ontario Veterinary College, University of Guelph, Guelph, Ontario Canada; Centre for Food-borne, Environmental and Zoonotic Infectious Diseases, Public Health Agency of Canada, 120-255 Woodlawn Road West, Guelph, Ontario Canada; Fraser Health, Suite 400, Central City Tower, 13450-102nd Avenue, Surrey, V3T 0H1 British Columbia UK

**Keywords:** Source attribution, Expert opinion, Environmental health officer, Enteric disease, British Columbia, FoodNet Canada

## Abstract

**Background:**

Policies and programs are needed to mitigate the burden of enteric disease in Canada. Source attribution, a goal of FoodNet Canada, can inform such strategies and can be accomplished with the information provided by expert opinion. This includes environmental health officers’ (EHOs) opinions on the “most likely source of infection” (MLSI) of confirmed cases of enteric disease that are investigated by the Fraser Health Authority in British Columbia, FoodNet Canada’s second sentinel site.

**Methods:**

Exposure data from the MLSI were categorized into ten groups and summarized for five enteric disease groups using endemic cases in the first analysis, and a combination of endemic and international travel cases for the second analysis. An exploratory analysis was also conducted on risk setting information in the MLSI. The final analysis involved using a logistic regression model (Wald test) to describe the inherent biases in the data.

**Results:**

Exposure proportions, by disease group, were similar to those of an analysis of MLSI data from FoodNet Canada’s Ontario sentinel site. Food exposure represented the greatest proportion of overall enteric disease (32.0%), as well as for salmonellosis (45.0%), verotoxigenic *E. coli* (VTEC) infection (38.1%), and campylobacteriosis (30.0%) cases. The majority of parasitic diseases (41.2%) were attributed to water exposure. Food safety practices and consuming unpasteurized products were more frequently reported for campylobacteriosis (19.7% and 5.4%, respectively) compared to other enteric diseases. More VTEC infection was attributed to domestic travel (4.8%) than the other enteric diseases. Among endemic and international travel-related cases combined, VTEC infection was attributed more to endemic food exposure (35.5%) than international travel (16.1%), but similar proportions of campylobacteriosis were attributed to endemic food exposure (25.1%) and international travel (25.1%). Variations existed in the exposure and risk setting information that EHOs included in the MLSI, and in their propensity to enter food sources over other types of exposures.

**Conclusions:**

Results from the MLSI analysis for exposure, risk setting, and EHO bias, are valid contributions for informing source attribution. Important considerations from this work, including strategies to standardize and improve the quality of MLSI data, will enhance source attribution hypotheses.

## Background

Enteric disease remains a significant public health issue in Canada. Without taking into consideration other exposures, it is estimated that 1 in 8 Canadians experience foodborne illness annually, with acute gastroenteritis costing approximately $3.7 billion a year [1, 2]. In British Columbia (BC), approximately 400,000 cases of acute gastroenteritis occur monthly with an annual cost of $514.2 million, and lost productivity accounting for 50.3% of the total cost [3, 4].

Source attribution is the alignment of sources of infection, for example animal reservoirs, food sources, person-to-person transmission, and water or environmental sources, with specific enteric diseases [5]. Quantifying the importance of potential transmission routes, through enhanced surveillance such as that conducted by FoodNet Canada, helps inform and prioritize policies to reduce the burden of enteric disease in Canada. Estimating source attribution for enteric disease can involve the use of multiple and complementary techniques including microbial sub-typing comparison, comparative exposure assessment, outbreak data analysis, case–control studies, intervention studies and expert elicitation [5, 6]. Expert elicitation, in particular, is considered a useful resource in source attribution, especially when data is lacking [5, 7–9].

In Canada, environmental health officers (EHOs), public health inspectors (PHIs), or public health nurses collect risk factor information from confirmed cases of gastroenteritis within their respective local jurisdictions. Studies in Ontario (ON) have looked at using the data collected as a means of harnessing PHI’s^a^ opinions for source attribution with respect to specific exposures [10–17], and the settings within which these exposures occurred [11, 13–17].

FoodNet Canada’s sentinel sites in ON and BC use an enhanced standardized questionnaire to interview reported cases of enteric disease. Within the questionnaire there is an open text question to capture the “most likely source of infection” or MLSI. This data element allows EHOs^b^ to provide their expert opinion as to what they think was a case’s most probable source of infection. In 2012, Dumoulin et al. explored the MLSI question using FoodNet Canada’s Ontario sentinel site (ON site) data [18]. The purpose of the current study was to analyze data from FoodNet Canada’s British Columbia sentinel site (BC site), to further explore its usefulness in informing source attribution in Canada. In addition, comparisons of endemic and travel-related sources of infection were made, risk setting information was extracted from the MLSI responses for exploratory purposes, and a brief analysis of EHO biases was conducted as a means of addressing the inherent bias of this source of data.

## Methods

### Study population, timeframe and design

The study population was FoodNet Canada’s BC site, within the Fraser Health Authority (FHA). The BC site comprises the communities of Burnaby, Abbotsford, and Chilliwack of the FHA, and has approximately 466,000 residents [19]. It was officially established as FoodNet Canada’s second sentinel site in April 2010. Depersonalized data from April 2010 to December 2012, acquired through a data sharing agreement, was included in the analysis.

### Exposure analysis

Exposure frequencies were analyzed for two subsets of cases: endemic cases with recorded MLSIs (subset A), and all endemic and international travel-related cases combined (subset B). Each subset was analyzed by the following five disease groups: i) overall enteric disease (campylobacteriosis, cryptosporidiosis, giardiasis, listeriosis, salmonellosis, shigellosis, verotoxigenic *Escherichia coli* (VTEC) infection, and yersiniosis), ii) campylobacteriosis, iii) salmonellosis, iv) VTEC infection, and v) parasitic diseases (cryptosporidiosis and giardiasis).

### Endemic cases (Subset A)

The methodology for this analysis was based on that described by Dumoulin et al., with a few modifications outlined herein [18]. Endemic cases included individuals who had an infection that was considered sporadic and domestically acquired (i.e. within Canada). International travel, outbreak, and lost to follow-up cases were excluded from subset A. International travel cases were defined as those who acquired their illness due to an exposure that occurred outside of Canada, i.e. the responsible pathogen’s incubation period fell within the period that the case was abroad. An EHO may still consider an exposure that occurs abroad but falls outside the incubation period of a pathogen as a feasible source of infection. As a result, some endemic cases had an international travel or immigration-related exposure recorded in the MLSI; these cases were therefore excluded from subset A. Endemic cases with MLSI information that was missing or unknown were removed.

The remaining endemic cases with MLSI entries were included in the analysis and were classified using at least one of ten exposure categories: i) water, ii) food, iii) food safety practices, iv) unpasteurized, v) occupational, vi) environmental, vii) animal, viii) person-to-person, ix) domestic travel, and x) other (Table 1). EHOs who concluded more than one possible exposure for a case resulted in multiple classifications for one case.

**Table 1:** **Guidelines for the classification of MLSI entries by exposure for BC site (2010–2012)**

MLSI exposure classification	Definition
Water	Consumption or physical contact with water through activities such as swimming
Food	Any beverage or food item for human consumption
	***Includes:***
	Spoiled/contaminated food
	Food events in which consumption of food or beverages is assumed, such as at a restaurant or Christmas party
	Food items intended to be consumed undercooked (except chicken and raw clams), such as sushi, runny eggs, raw eggs and raw beef
	***Excludes:***
	Exposure classified as unpasteurized or water
	Contaminated food whereby food safety practices are neglected
Food safety practices	Unsafe food safety practices including issues regarding food handling, food preparation, hand hygiene, cross-contamination, temperature (e.g. undercooking), and thawing meat
	***Excludes:***
	Exposures related to work/occupation
	Cross-contamination specifically stated from live animal exposure or person-to-person transmission
	Hand washing by a caregiver not specifically stated to be food related
Unpasteurized	Consumption of unpasteurized, raw or untreated milk, juice, or cheese
Occupational	Implied or directly stated work/occupational exposure
	***Excludes:***
	Specifically stated raw water exposure; this was classified as water
	Food consumed at work
Environmental	Exposure from the external physical environment, such as exposure to animal feces, animal remains, pet food, sewage backup, indirect animal contact (including eggs), and farm contact
	***Includes:***
	Activities such as camping hiking, playing in the park, or visiting a pumpkin patch
	Cross-contamination that does not involve food
	***Excludes:***
	Exposure classified as water, food, food safety practices, unpasteurized, occupational, animal or person-to-person
Animal	Potential animal contact
	***Excludes:***
	Contact through work/occupational exposure
	Farm/barn contact without specifically stated animal contact/exposure; this was classified as environmental
Person-to-person	Implied or directly stated person-to-person transmission
	***Includes:***
	Cases born with listeriosis
	Acquired through sexual transmission
	Acquired through breastfeeding
	***Excludes:***
	Contact through work/occupational exposure
Domestic travel	Exposure during travel outside Fraser Health Services Delivery Areas plus areas 38, 161, 162, 163, 164, 165, 166, 44, and 45 [20]
Other	MLSI that did not fit in above eight categories
	***Includes:***
	Exposures stated as settings (or contact with) such as household, home, long-term care facility, hospital, day care, support care facility, and short-term stay shelter
	Chronic/asymptomatic carriers

Adapted from Dumoulin et al. [18].

The category ‘domestic travel’ was created specifically for the BC sentinel site. FHA considers exposures that occur outside the geographical boundaries of the Fraser Health Authority and adjacent portions of Vancouver-Coastal Health Authority to be domestic travel. Cases where the EHO explicitly stated that an exposure occurred during domestic travel, such as “food while travelling”, “travel within BC”, or “Saskatchewan” were categorized as ‘domestic travel’.

Referring to case risk factor information collected from the questionnaire to better interpret the MLSI was generally avoided to maintain the integrity of the study. However, there were exceptions to this rule. For example, the MLSI category ‘occupational’ was defined based on explicit statements in the MLSI field, such as “work at chicken farms…”, and implicit statements, for example “contact with chickens/farm animals”, where case risk factor data was consulted to confirm that such implicit statements were in fact referring to occupational exposures. Also, in some instances the pathogen responsible for a case’s illness was referred to for insight. For example, the MLSI entry “born with the illness” was categorized as ‘person-to-person’ once it was confirmed that the case was a listeriosis case.

Entries that described a setting such as “home”, “day care” or “long-term care facility” were classified as ‘other’ because such settings provide the opportunity for various, unspecified exposures. As a result, in entries that included a setting and an associated source separated by a divider (“or”, semi-colon, slash or a comma), the setting was ignored and only the specified source was categorized. For example, “household, bbq chicken or steak, handle raw meat” was categorized as ‘food’ and ‘food safety practices’, and not as ‘other’.

The category ‘food safety practices’ was generally used for sources of infection that involved unsafe food preparation and cooking, for example undercooking food items. Undercooked eggs were considered an exception in the classification of food safety practices because the consumption of undercooked or raw eggs was considered to be a matter of food consumption preference and were therefore classified under ‘food’. If “eggs” or “chicken” were included in the MLSI without further indication of whether the exposure was food-related, environment-related or animal-related, the source of infection was classified as ‘food’.

### Endemic and international travel cases (Subset B)

International travel-related enteric disease cases were included in subset B as well as all the endemic cases from subset A. Endemic cases that had an international travel or immigration-related exposure recorded in the MLSI were classified as ‘international travel’ in subset B. The original ten exposure categories from subset A were collapsed into five categories. The results were subsequently summarized using these five categories plus two additional categories, ‘unknown’ and ‘international travel’. Therefore, the exposure categories used in this analysis were: i) unknown (includes cases with a missing or unknown MLSI), ii) water, iii) food (includes the categories food, food safety practices, and unpasteurized from subset A), iv) animal, v) person-to-person, vi) other (includes the categories occupational, environmental, domestic travel, and other from subset A), and vii) international travel.

### Risk setting analysis

Cases from subset A were also included in a risk setting analysis. The open text content of the MLSI was classified using nine setting categories: i) private home, ii) food premises, iii) farm, iv) recreational, v) institutional, vi) workplace, vii) domestic travel, viii) other, and ix) unclear (Table 2).

**Table 2:** **Guidelines for the classification of MLSI entries by risk setting for BC site (2010–2012)**

MLSI risk setting classification	Definition
Private home	Exposure occurred in a private home
	***Includes:***
	Home-prepared food
Food premises	Any place where food intended for public consumption is sold, offered for sale, supplied, handled, prepared, packaged, displayed, served, processed, stored, transported or dispensed [21]
	***Includes***
	Popular expressions for food offered at establishments; “Chinese food” or “food item from Korean BBQ”
Farm	Farm and farm animal exposure
	***Excludes:***
	Exposures that are related to an occupation
	Exposures that are explicitly stated to have occurred at home e.g. “home farm”
Recreational	Local travel: petting zoo; pumpkin patch or park; activities including hiking, camping, hunting, recreational water contact or gardening; or exposure to lakes/rivers/creeks
	***Excludes:***
	Exposures that are explicitly stated to have occurred at home e.g. “gardening at home”; this should be classified as private home
	Food exposure during recreational activity e.g. food at a fair
Institutional	Long-term care facility, hospital, day care, support care facility
Workplace	Implied or directly stated work/occupational exposure
	***Includes:***
	Food consumed at work
Domestic travel	Exposure during travel outside Fraser Health Services Delivery Areas plus areas 38, 161, 162, 163, 164, 165, 166, 44, and 45 [20]
Other	Short-term stay shelter
Unclear	Where setting for an MLSI is not provided or is ambiguous

The classification ‘unclear’ was used for entries where none of the possible sources of infection had obvious risk settings. For example, “animal contact, game meat, or sick contact” was classified as ‘unclear’ since none of the three exposures provided in the MLSI field were associated with identifiable risk settings. Any entries that were classified as ‘occupational’ or ‘domestic travel’ in the exposure analysis were subsequently classified as ‘workplace’ or ‘domestic travel’, respectively. Entries were automatically categorized as ‘private home’ when the EHO stated that the source of infection was linked with a home or household setting, regardless of whether the setting matched other categories. For example, “gardening at home” was classified as ‘private home’ and not ‘recreational’. If it was specifically stated that “farm animal” or “farm” exposure occurred, the MLSI was classified as ‘farm’, however assumptions of farm exposure were not made in entries such as “raw milk or cattle exposure”, which was subsequently categorized as ‘unclear’.

### EHO biases analysis

Data from subset A was used in a logistic regression model to identify any potential biases present in the EHOs’ opinions that may systematically implicate certain sources of infection. The EHOs responsible for interviewing cases in FHA were anonymized and coded numerically from one to nine. Cases where the MLSI was classified more than once were coded as ‘multiple’ and excluded. Cases with a single possible exposure source were coded according to the five exposure categories: i) water, ii) food (includes the categories food, food safety practices and unpasteurized from subset A), iii) animal, iv) person-to-person, and v) other (includes the categories occupational, environmental, domestic travel and other from subset A). These categories were further collapsed into the two exposure categories ‘food’ and ‘other’ for a dichotomous outcome of interest.

### Statistical analysis

All descriptive and statistical analyses were performed using SAS version 9.3 (SAS Institute Inc., Cary, NC, USA, 2002–2010). Frequency distributions for exposure categories by total exposure and by total number of cases, for each disease group, were tabulated for subset A and B. For the risk setting analysis, frequencies were tabulated for each setting category and presented in percent by total risk setting and percent by total number of cases for overall enteric disease.

A logistic regression model was developed to explore the statistical significance of EHOs choosing food over other exposure sources for overall enteric disease. Other factors were taken into consideration when developing this model, including the geographic residence of a case (Abbotsford, Burnaby, and Chilliwack) and season of disease onset (summer and not summer). These were important factors to consider in the analysis of EHO biases because an EHO may have interviewed more cases in certain geographic residences, and potential exposures change with the seasons, particularly in the summer when outdoor activities are more common.

First, EHO was run with the exposure variable without controlling for geographical or seasonal effects. The Wald test (p < 0.05) was used to determine the significance of each univariable model. Forward selection was then used to build the best model that included EHO as a predictor. Geography was then included in the model, followed by season, and finally geography and season together. The model with the greatest significance was then summarized by creating point estimates and 95% confidence intervals for the odds of each EHO choosing ‘food’ over ‘other’ compared to every other EHO, while controlling for the appropriate factors.

## Results

### Study population

A total of 1132 enteric disease cases under FoodNet Canada surveillance were reported to the BC site from April 2010 to December 2012: 12.5% (142) were lost to follow-up, 58.9% (667) were classified as endemic, 26.6% (301) were international travel-related, and 1.9% (22) were outbreak-related.

Of the 667 endemic cases in the full data set, 65.2% (435) had known possible sources of infection and were included in subset A (Table 3). A maximum of 3 separate exposures was recorded for the cases: 82.1% (357) of the cases had 1 possible source of infection, 16.3% (71) had 2 possible exposures, and 1.6% (7) had 3 potential sources of infection.

**Table 3:** **Summary of endemic cases excluded and included in the MLSI analysis – Subset A**

	Excluded MLSI cases			Included MLSI cases ^*^				Total endemic	% of total endemic
Disease	Missing	Immigration + international travel	Unknown	Unknown plus text ^†^	Known	Total included	% of total included		
Campylobacteriosis	1	0	100	20	183	203	46.7	304	45.6
Cryptosporidiosis	0	0	4	0	7	7	1.6	11	1.6
Giardiasis	0	10	21	2	42	44	10.1	75	11.2
Listeriosis	0	0	2	0	2	2	0.5	4	0.6
Salmonellosis	1	2	52	4	127	131	30.1	186	27.9
Shigellosis	0	1	3	0	8	8	1.8	12	1.8
VTEC infection	0	1	5	0	21	21	4.8	27	4.0
Yersiniosis	0	0	29	1	18	19	4.4	48	7.2
**Total**	2	14	216	27	408	435		667	
**% of total endemic**	0.3	2.1	32.4	4.0	61.2	65.2			100.0

^*^Water, Animal, Food, Food safety practices, Environmental, Occupational, Unpasteurized, Person-to-person, Other and, Domestic travel classifications.

^†^Entered as ‘unknown’ but also included exposure information.

Subset B had 961 enteric disease cases of which 45.3% (435) were endemic cases with known MLSIs, 22.7% (218) were endemic cases with unknown or missing MLSIs, and 32.0% (308) were international travel-related cases (included endemic cases with a recorded international travel or immigration-related MLSI). Unknown and missing MLSI entries were included in this analysis to produce a denominator that represented a true ratio of endemic to international travel cases in the sample.

### Exposure analysis

#### Endemic cases (Subset A)

Table 4 summarizes the results for each disease group. Exposure is presented as a percent of the total number of exposures—which counts cases more than once if there is more than one exposure—and also as a percent of the total number of cases. Food exposure represented the greatest proportion of overall enteric disease (32.0%), as well as for salmonellosis (45.0%), VTEC infection (38.1%), and campylobacteriosis (30.0%) cases. Only 3.9% of parasitic diseases were attributed to food. Food safety practices presented an important source for campylobacteriosis (19.7%) compared with the other disease groups. The ‘unpasteurized’ category appeared most frequently for campylobacteriosis (5.4%), followed by VTEC infection (4.8%) and overall enteric disease (3.0%). A very small proportion of salmonellosis cases were represented by ‘unpasteurized’ (0.8%) and no such exposure was recorded among parasitic diseases.

**Table 4:** **Summary of MLSI exposure classifications for endemic cases – Subset A**

Disease group	Food	Water	Food safety practices	Unpasteurized	Occupational	Environmental	Animal	Person-to-person	Other	Domestic travel
**Campylobacteriosis**										
Frequency of MLSI exposure	61	15	40	11	31	22	34	4	31	8
% by exposure (257)	23.7	5.8	15.6	4.3	12.1	8.6	13.2	1.6	12.1	3.1
% by number of cases (203)	30.0	7.4	19.7	5.4	15.3	10.8	16.7	2.0	15.3	3.9
**Salmonellosis**										
Frequency of MLSI exposure	59	3	12	1	4	12	14	11	27	2
% by exposure (145)	40.7	2.1	8.3	0.7	2.8	8.3	9.7	7.6	18.6	1.4
% by number of cases (131)	45.0	2.3	9.2	0.8	3.1	9.2	10.7	8.4	20.6	1.5
**VTEC infection**										
Frequency of MLSI exposure	8	1	2	1	1	1	4	1	5	1
% by exposure (25)	32.0	4.0	8.0	4.0	4.0	4.0	16.0	4.0	20.0	4.0
% by number of cases (21)	38.1	4.8	9.5	4.8	4.8	4.8	19.0	4.8	23.8	4.8
**Parasitic diseases** ^*^										
Frequency of MLSI exposure	2	21	0	0	6	9	5	8	6	2
% by exposure (59)	3.4	35.6	0.0	0.0	10.2	15.3	8.5	13.6	10.2	3.4
% by number of cases (51)	3.9	41.2	0.0	0.0	11.8	17.6	9.8	15.7	11.8	3.9
**Overall enteric disease** ^†^										
Frequency of MLSI exposure	139	41	54	13	43	46	58	33	80	13
% by exposure (520)	26.7	7.9	10.4	2.5	8.3	8.8	11.2	6.3	15.4	2.5
% by number of cases (435)	32.0	9.4	12.4	3.0	9.9	10.6	13.3	7.6	18.4	3.0

Note: some cases had more than one exposure in MLSI.

^*^Cryptosporidiosis and Giardiasis.

^†^Campylobacteriosis, Cryptosporidiosis, Giardiasis, Listeriosis, Salmonellosis, Shigellosis, VTEC infection, and Yersiniosis.

The majority of parasitic diseases (41.2%) were attributed to water exposure, with water occurring most frequently in this disease group compared to other enteric diseases. For overall enteric disease, salmonellosis, campylobacteriosis, and VTEC infection, water exposure was less common and ranged from 2.3 to 9.4%. Person-to-person exposure was also recorded more frequently for parasitic diseases (15.7%) compared to the other diseases.

Animal contact was the third most important exposure source for VTEC infection (19.0%), campylobacteriosis (16.7%), overall enteric disease (13.3%), and salmonellosis (10.7%); and represented a less common exposure for parasitic diseases (9.8%).

Occupational exposure was most common in campylobacteriosis cases (15.3%), followed by parasitic diseases (11.8%) and overall enteric disease (9.9%). Environmental exposure was the second most important exposure for parasitic diseases and recorded most frequently for this disease group (17.6%) compared to the other diseases. The category ‘other’ comprised a significant proportion of all diseases, from 11.8 to 23.8% of the cases. Finally, more VTEC infection was attributed to domestic travel exposure (4.8%) than the other disease groups which varied between 1.5 to 3.9%.

#### Endemic and international travel cases (Subset B)

Table 5 provides a summary of the results for the analysis of subset B for each disease group, by total exposure and by total number of cases. International travel represented almost half (46.5%) of the exposures among parasitic disease cases. The same proportion of campylobacteriosis cases was attributed to endemic food exposure as international travel (25.1%), while VTEC infection was attributed more to endemic food exposure (35.5%) than to international travel (16.1%).

**Table 5:** **Summary of MLSI exposure classifications for endemic and international travel cases – Subset B**

Disease group	Unknown ^‡^	Water	Food ^§^	Animal	Person-to-person	Other ^***║***^	International travel
**Campylobacteriosis**							
Frequency of MLSI exposure	101	15	102	34	4	91	102
% by exposure (449)	22.5	3.3	22.7	7.6	0.9	20.3	22.7
% by number of cases (406)	24.9	3.7	25.1	8.4	1.0	22.4	25.1
**Salmonellosis**							
Frequency of MLSI exposure	53	3	68	14	11	44	106
% by exposure (299)	17.7	1.0	22.7	4.7	3.7	14.7	35.5
% by number of cases (290)	18.3	1.0	23.4	4.8	3.8	15.2	36.6
**VTEC infection**							
Frequency of MLSI exposure	5	1	11	4	1	8	5
% by exposure (35)	14.3	2.9	31.4	11.4	2.9	22.9	14.3
% by number of cases (31)	16.1	3.2	35.5	12.9	3.2	25.8	16.1
**Parasitic diseases** ^*^							
Frequency of MLSI exposure	25	21	2	5	8	22	66
% by exposure (149)	16.8	14.1	1.3	3.4	5.4	14.8	44.3
% by number of cases (142)	17.6	14.8	1.4	3.5	5.6	15.5	46.5
**Overall enteric disease** ^**†**^							
Frequency of MLSI exposure	218	41	192	58	33	179	308
% by exposure (1029)	21.2	4.0	18.7	5.6	3.2	17.4	29.9
% by number of cases (961)	22.7	4.3	20.0	6.0	3.4	18.6	32.0

Note: some cases had more than one exposure in MLSI.

^*^Cryptosporidiosis and Giardiasis.

^†^Campylobacteriosis, Cryptosporidiosis, Giardiasis, Listeriosis, Salmonellosis, Shigellosis, VTEC infection, and Yersiniosis.

^‡^Missing or unknown MLSI.

^§^Food, Food safety practices, and Unpasteurized.

^*║*^Occupational, Environmental, Domestic travel, and Other.

### Risk setting analysis

Table 6 provides a summary of the analysis of subset A for risk setting, presented as a percent of the total number of settings - which counts cases more than once if there is more than one risk setting - and as a percent of the total number of cases for overall enteric disease. Risk setting was not summarized by disease group due to the large proportion of MLSI entries with missing data.

**Table 6:** **Summary of MLSI risk setting classifications for endemic cases – Subset A**

Disease group	Food premises	Private home	Farm	Recreational	Institutional	Workplace	Other	Domestic travel	Unclear
**Overall enteric disease** ^*^									
Frequency of MLSI risk setting	26	149	24	33	11	44	1	13	159
% by risk setting (460)	5.7	32.4	5.2	7.2	2.4	9.6	0.2	2.8	34.6
% by number of cases (435)	6.0	34.3	5.5	7.6	2.5	10.1	0.2	3.0	36.6

Note: some cases had more than one risk setting in MLSI.

^*^Campylobacteriosis, Cryptosporidiosis, Giardiasis, Listeriosis, Salmonellosis, Shigellosis, VTEC infection, and Yersiniosis.

A large proportion of the cases were classified as ‘unclear’ (36.6%) and ‘private home’ (34.3%). Cases were attributed to exposures in food premises approximately 6% of the time.

### EHO biases analysis

The initial logistic regression model using subset A with EHO as a predictor and exposure source as an outcome was marginally significant (Wald test, p = 0.0885). In the process of forward selection it was determined that the model including geography was significant (Wald test, p = 0.0002), while the model including season was not (Wald test, p = 0.1179). The inclusion of both factors (geography and season) resulted in a model that was significant (Wald test, p = 0.0003), but did not substantially change either the strength of the associations or the actual parameter estimates produced with the model that included geography alone. As a result, point estimate odds ratios, and associated 95% confidence intervals, were summarized for each EHO’s choice of ‘food’ over ‘other’ for overall enteric disease, in comparison to all other EHOs while controlling only for geography (Table 7).

**Table 7:** **Point estimate odds ratios (95% confidence interval) of EHOs choosing food over other – Subset A**

Referent	Overall enteric disease ^†^								
EHO	1	2	3	4	5	6	7	8	9
**1**		0.47 (0.22-1.00)*	0.45 (0.21-0.95)*	0.15 (0.06-0.35)*	1.28 (0.34-4.83)	0.52 (0.20-1.40)	0.62 (0.16-2.43)	0.68 (0.22-2.10)	0.31 (0.13-0.74)*
**2**	2.13 (1.00- 4.54)*		0.96 (0.47-1.94)	0.32 (0.17-0.61)*	2.74 (0.72-10.41)	1.12 (0.42-3.00)	1.32 (0.38-4.64)	1.45 (0.45-4.61)	0.66 (0.29-1.51)
**3**	2.22 (1.06-4.67)*	1.04 (0.52-2.11)		0.33 (0.15-0.72)*	2.85 (0.75-10.84)	1.17 (0.43-3.13)	1.38 (0.37-5.16)	1.51 (0.48-4.76)	0.69 (0.29-1.63)
**4**	6.65 (2.84-15.57)*	3.12 (1.63-5.96)*	2.99 (1.38-6.48)*		8.54 (2.14-34.12)*	3.49 (1.22-9.95)*	4.13 (1.17-14.52)*	4.51 (1.33-15.29)*	2.06 (0.85-4.96)
**5**	0.78 (0.21-2.93)	0.37 (0.10-1.39)	0.35 (0.09-1.33)	0.12 (0.03-0.47)*		0.41 (0.09-1.77)	0.48 (0.08-2.78)	0.53 (0.11-2.57)	0.24 (0.06-0.99)*
**6**	1.91 (0.72-5.08)	0.90 (0.33-2.40)	0.86 (0.32-2.30)	0.29 (0.10-0.82)*	2.45 (0.56-10.63)		1.18 (0.26-5.30)	1.29 (0.35-4.76)	0.59 (0.20-1.75)
**7**	1.61 (0.41-6.30)	0.76 (0.22-2.65)	0.73 (0.19-2.72)	0.24 (0.07-0.85)*	2.07 (0.36-11.92)	0.85 (0.19-3.79)		1.09 (0.22-5.54)	0.50 (0.13-1.99)
**8**	1.48 (0.48-4.58)	0.69 (0.22-2.21)	0.66 (0.21-2.10)	0.22 (0.07-0.75)*	1.90 (0.39-9.22)	0.77 (0.21-2.85)	0.92 (0.18-4.64)		0.46 (0.13-1.58)
**9**	3.23 (1.35-7.71)*	1.52 (0.66-3.46)	1.45 (0.62-3.44)	0.49 (0.20-1.17)	4.15 (1.01-16.98)*	1.69 (0.57-5.02)	2.00 (0.50-8.01)	2.19 (0.64-7.55)	

*Significant associations, p < 0.05.

^†^Campylobacteriosis, Cryptosporidiosis, Giardiasis, Listeriosis, Salmonellosis, Shigellosis, VTEC infection, and Yersiniosis.

The odds ratios tended to cluster in each referent group with the exception of a number of outliers. For example, when EHO 4 is used as a referent; EHOs 2, 3, 6, and to some degree 7 and 8 have roughly the same values ranging from 2.99 to 4.51, while EHOs 4, 1, and 5 are outliers with point estimate odds ratios of 1.00, 6.65, and 8.54, respectively. These outliers have particularly lower or higher odds of entering a certain type of exposure in the MLSI field compared to the other EHOs.

## Discussion

The feasibility of using the MLSI from FoodNet Canada case questionnaires to inform source attribution hypotheses for gastroenteritis has been illustrated previously [18]. The current study builds on this work by exploring other types of information that can be extracted from the MLSI data element, such as risk setting and reporting bias.

For endemic cases, similar exposure patterns were observed for the five disease groups when compared to previous findings in Ontario that used comparable methodologies [18]. Food represented the largest exposure source for overall enteric disease as well as for the three major bacterial infections including campylobacteriosis, salmonellosis and VTEC infection. Another recent Canadian study also identified food to be the primary exposure source for gastrointestinal diseases [17]. These findings suggest that continued efforts are needed along the farm-to-fork continuum to reduce foodborne pathogen transmission. Food safety practices and animal contact also represented important exposure sources for campylobacteriosis, salmonellosis and VTEC infection. Previous studies have also shown that unsafe food safety practices (i.e. eating undercooked chicken) and direct contact with animals are significant risk factors for major enteric pathogens and contribute substantially to the overall burden of enteric disease [17, 22, 23]. For parasitic diseases, water represented the largest exposure source, which was expected, as the more frequent transmission of *Giardia* and *Cryptosporidium* via water over other exposures has been well documented [24, 25].

Slight discrepancies between the results from this study and those previously published by Dumoulin et al. using data from FoodNet Canada’s ON site are likely explained by differences between the two sentinel sites related to the EHOs’ data collection methods, the study population, geography, case numbers, disease types and MLSI categories (domestic travel exclusive to BC site analysis) [18]. The most notable difference was in the proportion of cases that were classified as ‘other’ in the exposure analysis, which was substantially higher for the BC site across all disease groups compared to the ON site. Of those BC cases, a large proportion of the MLSI entries included the terms ‘household’ and ‘home’, while fewer entries included long-term care facility or hospital settings. A review of the data indicated that a number of EHOs tended to enter a risk setting as the MLSI rather than an exposure. This tendency may be related to information requirements for BC’s Integrated Public Health Information System (iPHIS). iPHIS is an electronic database used by public health units to record information on reportable disease cases [26]. When an EHO completes a form in iPHIS they are asked to record a case’s potential exposure based on risk settings for example household, long-term care facility, or school. As a result, some EHOs may be completing the MLSI in a similar manner.

Domestic travel (explicitly stated in the MLSI field) accounted for very few of the exposures for overall enteric disease. Some MLSIs implicitly indicated an exposure that occurred during domestic travel, for example “drinking creek water while hiking”. Domestic exposure was confirmed in these instances using case risk factor information from the questionnaire. In total, there were 25 cases where domestic travel exposure was implicated, either implicitly or explicitly. EHOs only reported domestic travel exposure within the MLSI field for half of these cases. This observation indicates that EHOs may consider domestic travel to be a risk setting rather than an exposure in itself and consequently recorded the exposure without providing information about where the exposure may have occurred. A similar pattern was observed for MLSI entries with occupational exposures. For example, the following MLSI entry, “work at chicken farms in Chilliwack”, explicitly indicates occupational exposure; while in another MLSI entry, “contact with chickens/farm animals”, occupational exposure is not explicitly stated but was a confirmed exposure after consulting the case questionnaire.

International travel represented a substantial exposure for overall enteric disease cases in this study, and especially among parasitic disease cases where many of them reported travel related to recent immigration. In comparison, a study conducted by Taylor et al. attributed 31.7% of enteric diseases, for an urban population in BC, to confirmed international travel exposure [27]. This statistic indicates the importance of distinguishing between endemic and international travel cases for source attribution.

There are variations in how EHOs record their responses for the MLSI, particularly with regard to specifying the risk setting within which an exposure occurred. In order to encourage standardization of the type of information included in the MLSI field, it is suggested that EHOs be provided with clarification that the MLSI should include two components: a specific exposure and a risk setting. The EHO might also be asked to indicate on a scale of one to five how strongly they feel about their response, which could elucidate any uncertainties in the data. Such guidelines could be developed in order to increase the efficacy of using the data for source attribution while making sure that the EHO’s intuition is still reflected in the MLSI.

An inherent aspect of MLSI data, being opinion-based, is EHO response bias. An EHO might be more likely to attribute an enteric disease to one source over another due to professional experiences or recent publicized events including reported outbreaks or food recalls. In addition, the number of years of experience and interviewing skills can influence an EHO’s expert opinion and increase or decrease their odds of entering a certain type of exposure in the MLSI field compared to other EHOs. The results from this study demonstrate that the odds of choosing food over another exposure were roughly the same for five out of the nine EHOs, which indicates some degree of consistency. However, in addition to the above mentioned factors, the presence of outliers may also be related to selection bias as some EHOs may be assigned to cases that are more or less likely related to food. For example, EHO 4 had the lowest odds of choosing a food exposure over any other exposure compared to seven other EHOs, which may be related to the fact that this EHO had interviewed substantially fewer campylobacteriosis cases than three other EHOs and had interviewed the most number of giardiasis cases, an enteric disease often associated with non-food exposures [11, 24]. More years of data would allow for an analysis of EHO response choices using more exposure categories (i.e. food, water, animal etc.) for specific disease groups. Such an in-depth analysis could confirm where biases exist in the MLSI data. Additionally, the use of complementary EHO focus groups could provide a better understanding of what influences an EHO’s opinion. A final consideration could be to strengthen existing quality control protocols or adapting existing quality assurance programs such as those that periodically allow for dual observation and reporting. This may offer a clearer indication of any EHO biases that need to be addressed for MLSI interpretation.

Although an EHO determines an MLSI based on information collected from a standardized follow-up questionnaire, the strength of the MLSI comes from professional insight which may differ from responses to risk factor questions from the questionnaire. A future analysis could compare questionnaire risk factor data to MLSI exposure data in order to determine how the results corroborate.

### Limitations

Subjective interpretation of the MLSI responses must always be considered when making conclusions about exposures that are important for different disease groups. A mitigation strategy for such bias could involve the development of a continuously improved methodology for analysts to use in the interpretation of MLSI data between sentinel sites, to ensure a standardized procedure. For example, using two people to categorize the data might limit such subjective bias.

Finally, the prevalence of risk setting in the MLSI field prompted an exploration of this aspect of the data. After classifying all of the MLSI entries for risk setting it became apparent that the results for this analysis had to be interpreted with caution, as many entries were incomplete and classified as ‘unclear’. However, these results were expected since the primary purpose of the MLSI is to collect exposure information. Training on clarification of exposure and risk setting and the inclusion of both entries in the MLSI field would provide more complete and comprehensive exposure and risk setting data and allow for a more robust analysis of risk setting.

## Conclusions

The most likely source of infection (MLSI) provides useful information for informing source attribution hypotheses, and for identifying differences and similarities of exposure patterns across diseases. A number of recommendations are made to inform strategies for the standardization and improvement of MLSI data quality, without compromising the data element’s capability of capturing EHOs’ opinions.

Suggestions are made to train and encourage EHOs to include both exposure and risk setting in the MLSI field, as well as to measure how confident EHOs are with their responses. The analysis of a larger data set would improve our understanding of existing biases for more specific exposures within disease groups. In addition, interpretation of MLSI results could be improved through EHO focus groups and by strengthening existing or implementing new quality assurance programs for enteric disease investigation and reporting. Future analysis comparing risk factor data from the case-follow up questionnaire with MLSI data could assist in validating the use of the MLSI method for generating source attribution hypotheses, as well as confirm or highlight important risk factors of enteric disease to inform public health policy and practice at all levels of government.

## Endnote

^a^Inspectors of public health in ON are referred to as PHIs; b. Inspectors of public health in BC are referred to as EHOs, and will be referred to as such in this article since the data was collected in BC.

## Abbreviations

BC: British Columbia
BC site: British Columbia sentinel site
EHO: Environmental health officer
FHA: Fraser health authority
iPHIS: Integrated public health information system
MLSI: “most likely source of infection”
ON: Ontario
ON site: Ontario sentinel site
PHI: Public health inspector
VTEC: Verotoxigenic *Escherichia coli* .
